# On Latent Pneumonia

**Published:** 1847-09

**Authors:** 


*On Latent Pneumonia.* By M. Saucerotte.—Although the latent form of pneumonia is not unknown to practitioners, it has not attracted so much attention as it deserves. In fact, until the work of Grisolle and a thesis by M. Raymond appeared, we had not any monograph on the subject. As it is, no observer, according to M. Saucerotte, has noticed the connection of the peculiar character of the disease (latency) with a certain epidemic constitution, but it has been customary to regard that character as depending either upon the obscurity of the signs furnished by auscultation and percussion, or upon the negligence or ignorance of the attendant. It is, however, certain that there is a form of pneumonia, which does not present either the ordinary symptoms or course of that affection, and which commences, so to speak, by the second stage, or that of hepatization.

With these introductory remarks, the author proceeds to narrate several cases, from which he deduces the following general history of the disease:

1. *Premonitory Symptoms* are either entirely absent or of slight importance, consisting in a general feeling of lassitude, with shivering, loss of appetite, and but little fever.

2. *Symptoms.*—The temperature of the skin is not sensibly augmented; the pungent heat of ordinary pneumonia is seldom or never present; and the pulse is usually but little affected. The respiration is natural, and the patient does not complain of pain in the chest. Percussion always elicits a dull sound over a considerable extent, and bronchial respiration is audible over the same locality. In some cases slight creptation may be heard around the hepatized spot.

3. *Progress and Duration.*—The duration of latent pneumonia is variable, and depends much on the constitutional powers of the individual, the means which have been employed, and other modifying circumstances. In some cases we have seen the disease linger for six or seven weeks. When the case terminates favorably, the dulness gradually disappears, and the bronchial souffle is replaced by crepitation, respiration becomes more free, and the general aspect of the patient improves.

4. *Diagnosis.*—Pleurisy is the affection with which latent phenomena are most likely to be confounded, and this error the author admits that he has often committed. In chronic pleurisy, however, there is constantly a pain in the side, and the region of the dulness varies with the position of the patient. Apoplexy and bulging of the intercostal spaces shortly clear up the diagnosis. The history of the case and the locality of the dulness are sufficient to draw a distinction between the disease in question and phthisis.

5. *Causes.*—For the most part, exposure to cold.

6. *Treatment.*—The complete absence of, or slight febrile disturbance attending this disease renders general bloodletting necessary. The author usually applied the cupping-glasses or leeches, followed by plasters. The only internal medicine mentioned by him is the tartarized antimony.—*Gazette Médicale.—Ibid.*

